# An Evaluation of Skylight Polarization Patterns for Navigation

**DOI:** 10.3390/s150305895

**Published:** 2015-03-10

**Authors:** Tao Ma, Xiaoping Hu, Lilian Zhang, Junxiang Lian, Xiaofeng He, Yujie Wang, Zhiwen Xian

**Affiliations:** College of Mechatronic Engineering and Automation, National University of Defense Technology, Changsha 410073, Hunan, China; E-Mails: tao_ma@live.com (T.M.); lilian-zhang@hotmail.com (L.Z.); jx_Lian@hotmail.com (J.L.); hexf_lit@126.com (X.H.); yjwang@nudt.edu.cn (Y.W.); xianzw2011@163.com (Z.X.)

**Keywords:** angle of polarization (AOP), degree of polarization (DOP), quantitative analysis, solar meridian, navigation

## Abstract

Skylight polarization provides a significant navigation cue for certain polarization-sensitive animals. However, the precision of the angle of polarization (AOP) of skylight for vehicle orientation is not clear. An evaluation of AOP must be performed before it is utilized. This paper reports an evaluation of AOP of skylight by measuring the skylight polarization patterns of clear and cloudy skies using a full-sky imaging polarimetry system. AOP measurements of skylight are compared with the pattern calculated by the single-scattering Rayleigh model and these differences are quantified. The relationship between the degree of polarization (DOP) and the deviation of AOP of skylight is thoroughly studied. Based on these, a solar meridian extracted method is presented. The results of experiments reveal that the DOP is a key parameter to indicate the accuracy of AOP measurements, and all the output solar meridian orientations extracted by our method in both clear and cloudy skies can achieve a high accuracy for vehicle orientation.

## 1. Introduction

Navigation is a critical and difficult issue in many applications for autonomous vehicles. Many animals can utilize the natural polarization patterns for navigation. It has provided inspiration for us. The desert ant *Cataglyphis bicolor*, for example, is able to utilize the skylight polarization pattern as a compass to forage for meters and return back to its nest on a straight line [1]. A range of marine animals, such as fishes and mollusks, can perceive the polarization of light, and use it for orientation and navigation [2]. Furthermore, dung-beetles can perceive the moonlight polarization patterns [3]. “Learning from nature” is a sagacious idea to solve navigation problems [4]. To date, there is rich literature about bio-inspired navigation sensors detecting the skylight polarization pattern [4,5,6,7,8,9,10]. In ground vehicle orientation, for example, a polarized light sensor measures the angle of polarization (AOP) of skylight which is perpendicular to the plane of scattering determined by the observer, the celestial point observed and the sun, according to the single-scattering Rayleigh model [11,12]. Then, we can obtain the vehicle direction with respect to the position of the sun. Combining this information with the azimuth of the sun, the vehicle heading can be determined. However, depending on the solar elevation and the cloud cover, the skylight polarization pattern differs more or less from the theoretical pattern calculated by the single-scattering Rayleigh atmosphere [7,11,12,13,14]. The error can significantly reduce the accuracy of the heading solution. Thus, the celestial polarization pattern must be evaluated prior to using of the AOP measurement of skylight. 

In practice, sky polarization patterns are not straightforward because of the complex and varying atmospheric conditions. Numerous theoretical and experimental investigations have focused on the subject [7,13,14,15,16,17]. Additionally, the underwater polarization patterns of skylight have been researched in many literatures [18,19,20,21,22,23]. Clear sky polarization patterns are generally consistent with the results calculated by the single-scattering Rayleigh theory apart from regions near the sun, anti-sun and neutral points [11,13]. Yet, in cloudy skies, the measured degree of polarization (DOP) can be largely reduced by the clouds compared with that of clear skies, and the AOP of skylight will also suffer some distortions when clouds are present [11,12,13]. In this case, the DOP pattern differs greatly from the single-scattering Rayleigh theory, and it is unreliable as orientation cue compared with the AOP pattern [13,24,25]; the AOP may be either parallel to the scattering plane (*i.e*., 90° from the Rayleigh sky case), in the same direction as the clear sky AOP, or undefined [12]. Furthermore, when the sun is hidden by thicker clouds, both DOP and AOP exhibit departures from that of the clear sky [11,12]. Consequently, patterns of AOP of skylight are not always perpendicular to the scattering plane, which is a thorny point for navigation. We must quantify these differences between AOP measurements and theoretical (single-scattering Rayleigh) patterns. However, quantitative analysis of these differences is scarce for vehicle orientation.

It is noticed that the aforementioned AOP patterns share a common characteristic that a deviated AOP is often accompanied with a low DOP. Per definition, the DOP is the measure of the proportion of light which is actually polarized to the total incident light [6]. Later on, we will prove that the lower the DOP is, the larger the error of the AOP measurement will be. Furthermore, crickets can perceive the polarized stimuli down to a DOP level of less than 7% [26], but crickets cannot perceive the skylight polarization patterns if DOP is lower than the threshold value 5% [11]. 

In this paper, we measured the polarization patterns of clear and cloudy skies by a CCD camera with a fish-eye lens, and compared the results with the corresponding theoretical patterns quantitatively. Especially, we conducted an in-depth study of the relationship between the value of the DOP and the departure of AOP. Based on the evaluation results, a solar meridian extracted method is presented. The experiment results show that DOP is a good parameter to indicate the accuracy of AOP measurements of skylight, and the maximum error of solar meridian orientations extracted by our method is less than 0.5° even on cloudy days. 

## 2. Materials and Methods

### 2.1. Instrument Description

**Figure 1:**
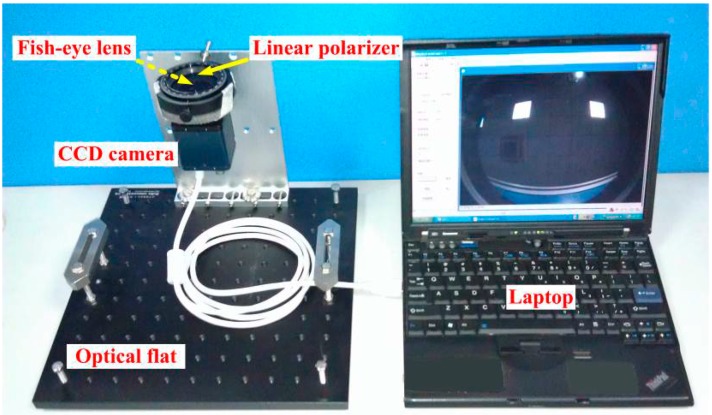
Full-sky imaging polarimetry system.

The full-sky imaging polarimetry system is shown in Figure 1. The technique used in this paper is similar to that of [13]. The full-sky imaging polarimetry mainly consists of a CCD camera (product model: KTJ-125M) equipped with a fish-eye lens (F-number = 1.6, focal length = 1.8 mm) and a manually rotated linear polarizer (product model: LPVISE200-A, diameter = 50.8 mm) which is in front of the fish-eye lens. As a result, the angle of view decreases from 180°–108°. All instruments are mounted on an optical flat (product model: OTSB33-1), which ensure the optical axes of the CCD camera, fish-eye lens and linear polarizer parallel to each other, and vertical pointing toward the zenith during the measuring process. Because the angle of view of the lens is colossal, the four-image technique is indispensable to improve the estimation accuracy of the skylight polarization patterns [27]. In this paper, we rotated the linear polarizer to four different relative orientations (0°, 45°, 90° and 135° clockwise from the top view of the camera) to obtain four digital images for the calculation of polarization patterns. The linear polarizer is fixed in an inner ring of a lens holder. The inner ring has a scale. Hence, we can rotate it to a certain position in the outer ring freely and accurately. 

### 2.2. Instrument Calibration

In this paper, we focus on the evaluation of AOP and DOP patterns of skylight, and the following two characteristics of the polarimetry system are taken into consideration in the calibration: (1) the measured reduced Mueller matrix of the fish-eye lens by the method in [25]; (2) the distortion of the fish-eye lens according to the calibration toolbox for Matlab [28,29]. 

It has been shown that the fish-eye lens has little effect on the light polarization patterns, which can be ignored in the measurement [13]. However, the fish-eye lens causes serious angle distortion on the incident light [29]. Consequently, we mainly calibrate the angle distortion caused by the fish-eye lens and the calibration results are listed in Table 1. The image size is 1280 × 960 pixels. 

**Table 1:** The calibration results.

Calibration Parameters (Pixel)	Values
Focal length ***f_c_***	[475.87278, 475.91533]
Principal point ***C_c_***	[643.71206, 477.27085]
Skew coefficient ***α_c_***	0.00047
Distortion coefficients ***k_c_***	[−0.26688, 0.07423, 0.00011, 0.00023, −0.00940]

### 2.3. Polarization Patterns Estimation and Analysis

In order to analyze the skylight polarization patterns, we calculate the AOP and DOP based on the Stokes polarization parameters. In general, light in the atmosphere is mostly linearly polarized, and the circularly polarized light is usually negligibly small [15,30], so the Stokes’ famous intensity formula can be simplified as Equation (1) [31].

(1) $$I\left(\text{α}\right)=\frac{1}{2}\left[I+Q\text{cos}2\text{α}+U\text{sin}2\text{α}\right]$$

where I, Q and *U* are Stokes parameters. I(α) is the intensity measured at a polarizer orientation angle α. To improve the estimation accuracy of the skylight polarization patterns, the three Stokes parameters are calculated by rotating the transmission axis of the polarizer to the angles α = 0°, 45°, 90° and 135°, respectively [16]. They can be obtained by Equation (2).

(2) $$\left[\begin{array}{c} I\\ Q\\ U \end{array}\right]=\left[\begin{array}{c} \left(I\left(0{}^{\circ}\right)+I\left(45{}^{\circ}\right)+I\left(90{}^{\circ}\right)+I\left(135{}^{\circ}\right)\right)/2\\ I\left(0{}^{\circ}\right)-I\left(90{}^{\circ}\right)\\ I\left(45{}^{\circ}\right)-I\left(135{}^{\circ}\right) \end{array}\right]$$

Then, the resulting degree of (linear) polarization *P* and its angle ψ are computed as Equations (3) and (4) [31]:

In general, the measurement errors of AOP patterns result from sensor manufacturing (scale factors, and offsets, *etc.*) and calibration procedure. However, we shall mathematically prove that the error of the AOP is also affected by the value of DOP. We therefore perform the variation operation on both sides of Equation (4). Then, we can get Equation (5):

(3) $$P=\frac{\sqrt{Q^{2}+U^{2}}}{I}$$

(4) $$\text{ψ}=\frac{1}{2}\text{arctan}\left|\frac{U}{Q}\right|$$

(5) $$\text{δψ}=\frac{-U}{2\left(Q^{2}+U^{2}\right)}\text{δ}Q+\frac{Q}{2\left(Q^{2}+U^{2}\right)}\text{δ}U$$

Let *U* = *IP*·sin 2ψ and, Q = *IP*·cos 2ψ so a simple form of Equation (5) is given by Equation (6).

(6) $$\text{δψ}=-\frac{\text{sin}2\text{ψ}}{2IP}\text{δ}Q+\frac{\text{cos}2\text{ψ}}{2IP}\text{δ}U$$

Note that the amplitudes of the coefficients of δQ and δ*U* decrease monotonically with the value of *P* for a given ψ and *I*; that is, other things being equal, a low DOP will result in a poor AOP measurement.

### 2.4. Using the Angle of Polarization (AOP) Patterns for Orientation

The polarization patterns of skylight can be measured by two methods: point-source based and image based [32]. The image-based method can obtain the polarization information of a region of the sky. Accordingly, we can use the AOP patterns of this region of the sky for orientation. According to the single-scattering Rayleigh model, one important feature of polarization information of a region with a center at the zenith is that the polarization pattern of skylight is symmetrical along the solar-antisolar meridian [13,25,33]. Hence, we can estimate the orientation of the solar meridian by detecting the symmetry axis of the polarization pattern of skylight. Combining the solar meridian orientation with the azimuth of the sun, we can robustly determine the vehicle heading. 

In the orientation process, the image based polarization sensor, such as our full-sky imaging polarimetry system, is aligned with the body frame (b-frame) of the vehicle. We assume that the polarization sensor frame (s-frame) coincides with b-frame. The s-frame consists of three orthogonal axes where the *x_s_*-axis is parallel with the row, *y_s_*-axis is parallel with the column of the pixels of the CCD sensor, respectively, and *z_s_*-axis is the optical axes pointing in the upward direction. The b-frame consists of three orthogonal axes where the *x*-axis points in the forward direction, *y_s_*-axis is in the direction of transverse motion, and *z*-axis is the upward direction. We let the *x* and *y* axis be parallel with the *x_s_* and *y_s_* axis, respectively. During the measuring process, we keep the *xy* plane horizontal; that is, the *z*-axis vertical points toward the zenith.

**Figure 2:**
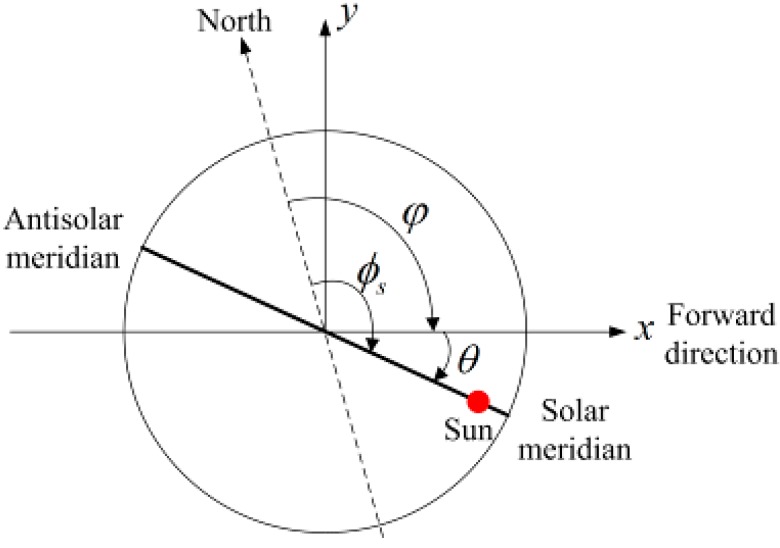
The relationship between the azimuth of the sun, the solar meridian orientation, and the vehicle heading in the horizontal plane.

Then, the angle between geographic north and the solar meridian in the *xy* plane denotes the azimuth of the sun $\phi_{s}$, the angle between the positive *x* axis and the solar meridian in the *xy* plane denotes the solar meridian orientation θ in the b-frame, and the angle between geographic north and the positive *x* axis denotes the vehicle heading φ Figure 2 shows the relationship among the three angles in the *xy* plane of the b-frame. 

Therefore, the vehicle heading φ can be determined by the following equation. We perform the variation operation on both sides of Equation (7), and we can get Equation (8).

(7) $$\text{φ}=\phi_{s}-\text{θ}$$

where $\phi_{s}$ can be obtained by the solar position calculation algorithm in [34] with an accuracy of 0.0083°, and θ can be solved by the AOP patterns measured by the polarization sensor.

(8) $$\text{δφ}=\text{δ}\phi_{s}-\text{δθ}$$

Neglecting the small value δΦ*_s_*, Equation (8) can be rewritten as Equation (9).

(9) δφ≈−δθ

Note that the accuracy of vehicle heading φ strongly depends on the solution precision of the solar meridian orientation θ. Hence, it is necessary to solve θ with a high accuracy. 

### 2.5. Solar Meridian Extracted from the Angle of Polarization (AOP) Patterns

**Figure 3:**
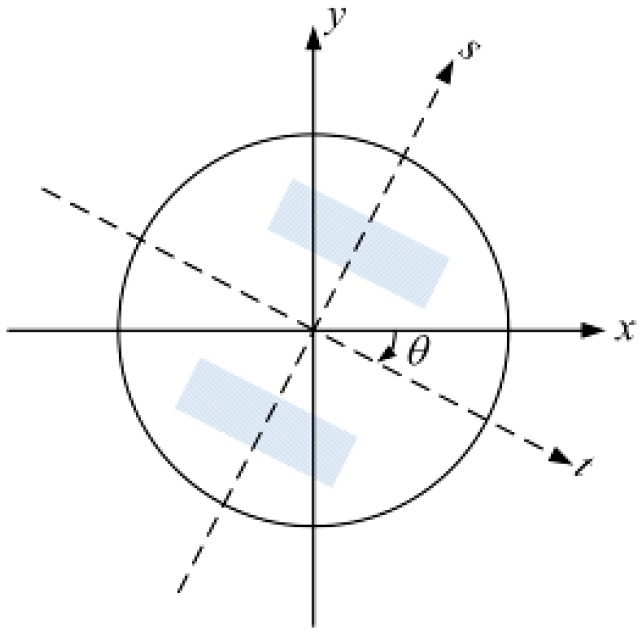
The coordinate system for the symmetry measurement.

Let ψ(*x*, *y*) be the AOP patterns of skylight measured by our polarimetry system, defined on a circle of radius *L* centered at the origin. Suppose *t* is the axis of symmetry of ψ(*x*, *y*) which passes through the origin and makes an angle θ with the positive *x* axis (Figure 3). It can be calculated as Equation (10). 

(10) $$\begin{array}{c} t=x\text{cosθ}-y\text{sinθ}\\ s=x\text{sinθ}+y\text{cosθ} \end{array}$$

The measurements are considered to be reflectionally symmetric if ψ(*t*, *s*) = ψ(*t*, −*s*) and antisymmetric if ψ(*t*, *s*) = −ψ(*t*, −*s*) [35]. Evidently, the AOP measurements ψ(*x*, *y*) is antisymmetric as expected from Rayleigh theory [29]. 

A measure of symmetry with respect to the *t* axis is computed as Equation (11) [35]:

(11) $$S_{\text{θ}}\left\{\text{ψ}\right\}=\frac{1}{2}\frac{\iint \text{ψ}\left(t,s\right)\text{ψ}\left(t,-s\right)dsdt}{\int {\text{ψ}}^{2}\left(t,s\right)dsdt}+\frac{1}{2}$$

where $S_{\text{θ}}\left\{\text{ψ}\right\}\in \left[0,\;1\right]$. Finding the minimum of $S_{\text{θ}}\left\{\text{ψ}\right\}$ parameterized by the axis *t*, we will get the only axis of symmetry of ψ(*x*, *y*), and this axis of symmetry is the orientation of solar and antisolar meridian. 

Note that the AOP patterns may suffer some distortions, for instance, when clouds appear. This has negative effects on the calculation of the measure of symmetry *S*_θ_ {ψ}. In order to solve this problem, we set all the AOP patterns to zero when the corresponding AOP deviations are large. The experimental results in Section 4.1 will show that a deviated AOP is often accompanied with a low DOP. Hence, we consider the AOP deviations are large when the corresponding DOP pattern is less than a DOP threshold. *P_threshold_* Note that crickets cannot perceive the skylight polarization patterns if DOP is lower than the threshold value 5% [11]. We use *P_threshold_* = 5% for our solar meridian extraction algorithm. Furthermore, the AOP measurements with the same distance from the origin as these points have also been set to zero. 

According to the Rayleigh theory, the absolute values of AOP measurements on the solar and antisolar meridian are equal to 90° [29]. Calculating the average slope of those points that satisfy the condition |ψ(x,y)|≈90°, we can obtain the rough value of the solar meridian orientation θ_0_. The solar meridian extraction method in [36] only uses the AOP measurements on the solar and antisolar meridian. If those points are distorted by the clouds, it will fail to obtain the solar meridian orientation. 

In order to obtain a more stable solar meridian orientation, a refinement method is presented in this paper. The procedures of our method are described as follows:

Step 1. Calculate the AOP measurements ψ(*x*, *y*) and the DOP measurements *P*(*x*, *y*) using Equations 2 and 3. 

Step 2. Set the AOP measurements to zero when the corresponding AOP deviations are large. The experimental results in Section 4.1 will show that a deviated AOP is often accompanied with a low DOP. Hence, we consider the AOP deviations are large when the corresponding DOP pattern is less than a DOP threshold *P_threshold_*. We use *P_threshold_* = 5% in the experiment.

Step 3. Set the AOP measurements with the same distance from the origin as these points in Step 2 to zero.

Step 4. Extract solar–antisolar meridian points in the AOP measurements ψ(x,y), where the absolute values of AOP are very close to 90°. Calculate the average slope of these points as the rough value of the solar meridian orientation θ_0_. 

Step 5. Calculate $S_{\text{θ}}\left\{\text{ψ}\right\}$ for different θ values (θ_1_, θ_2_, … , θ_*n*_) using Equation (11), where the value range of θ is set from ${\text{θ}}_{0}-\text{ε}$ to ${\text{θ}}_{0}+\text{ε}$. ε is a small positive number and *n* is a positive integer. We use ε = 2° and *n* = 10 in the experiment. Then, we can obtain a $S_{\text{θ}}\left\{\text{ψ}\right\}$ series $\left(S_{{\text{θ}}_{1}}\left\{\text{ψ}\right\},S_{{\text{θ}}_{2}}\left\{\text{ψ}\right\},\cdots ,S_{{\text{θ}}_{n}}\left\{\text{ψ}\right\}\right)$. 

Step 6. ${\text{θ}}_{i},\;i\in \left[1,\;n\right]$ can be selected as the accurate solar meridian orientation if and only if $S_{{\text{θ}}_{i}}\left\{\text{ψ}\right\}$ is the minimum value in the series $\left(S_{{\text{θ}}_{1}}\left\{\text{ψ}\right\},S_{{\text{θ}}_{2}}\left\{\text{ψ}\right\},\cdots ,S_{{\text{θ}}_{n}}\left\{\text{ψ}\right\}\right)$.

For convenience, we refer to this solar meridian extraction method as “our method”, and refer to the method not containing steps 2 and 3 as “our method without pretreatment” for distinguishing between them. 

## 3. Simulation Studies

In this section, simulations have been made to assess the performance of the proposed solar meridian extraction methods and compare them with the solar meridian extraction method in [36]. For convenience, we call this method the “fitting based method” in the following text. Assume that the solar elevation is 50° and the solar meridian orientation in the b-frame is −30°. Each map contains approximately 125,600 pixels. In order to simulate the noise involved in the AOP measurements, zero-mean white Gaussian noise is added to the simulation data. The standard variance of noise σ is set to 0, 1, 2 and 4 degrees, respectively. Through Monte Carlo simulations, we evaluate the performance of the algorithm by computing the mean error. 

**Figure 4:**
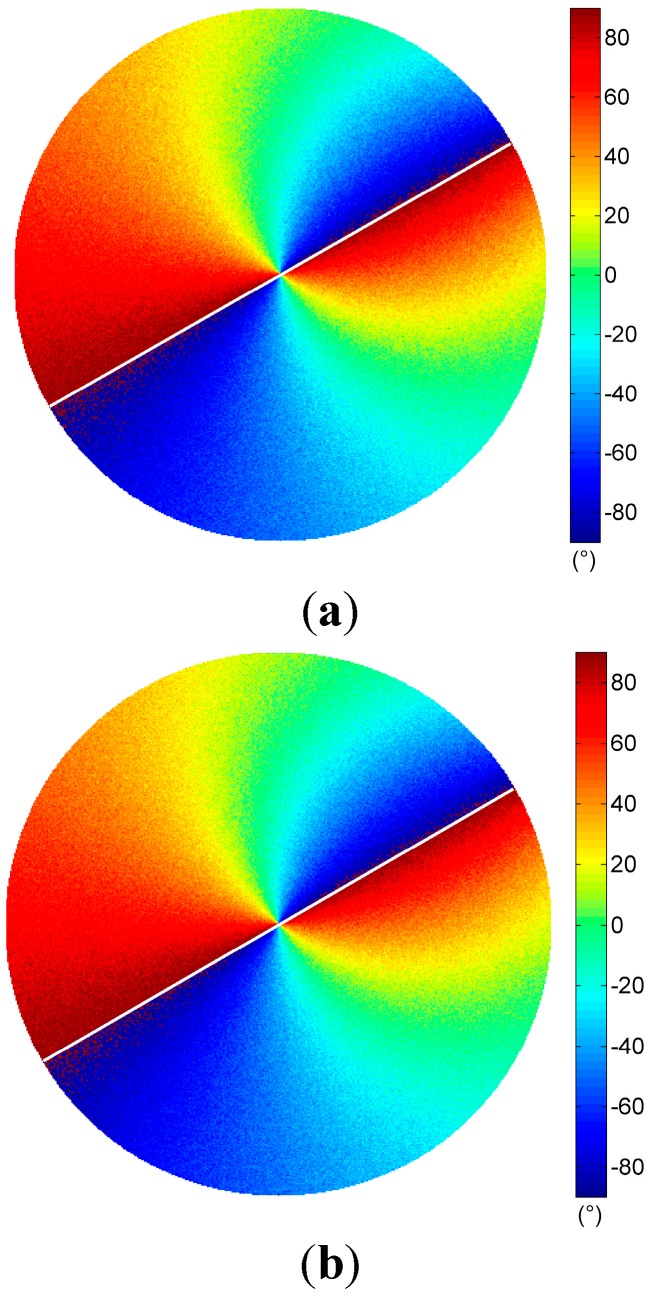

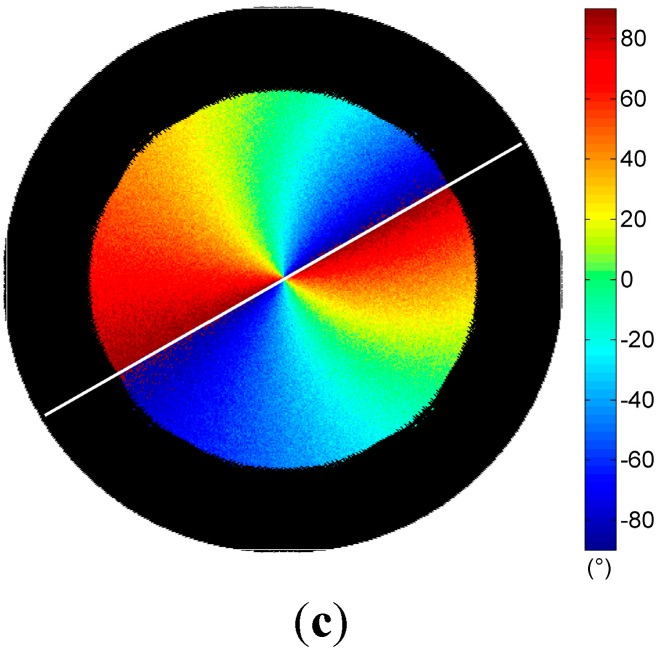
The solar meridian orientations extracted by (**a**) fitting based method; (**b**) our method without pretreatment and (**c**) our method when the standard variance of noise σ is 4 degrees.

Figure 4 contains the plots of the estimates for different methods when the standard variance of noise σ is 4 degree. The simulation results are summarized in Table 2. It can be seen from the table that our method performs slightly better than the fitting based method. All the methods can obtain stable estimates even under the condition that the standard variance of noise σ is 4 degrees in the AOP measurements. It is noticed that the fitting based method only use the AOP measurements on the solar and antisolar meridian. If those points are distorted by the clouds, the solar meridian orientation will not be obtained.

**Table 2:** The error of the solar meridian orientation extracted by the three methods

Measurement Noise	Calculation Methods	Mean Error (°)
No noise	Fitting based method	0.18
	Our method without pretreatment	0.15
	Our method	0.06
σ = 1°	Fitting based method	0.20
	Our method without pretreatment	0.32
	Our method	0.16
σ = 2°	Fitting based method	0.23
	Our method without pretreatment	0.36
	Our method	0.19
σ = 4°	Fitting based method	0.35
	Our method without pretreatment	0.37
	Our method	0.20

## 4. Experiment Results

### 4.1. Skylight Polarization Measurements and Evaluation

**Figure 5:**
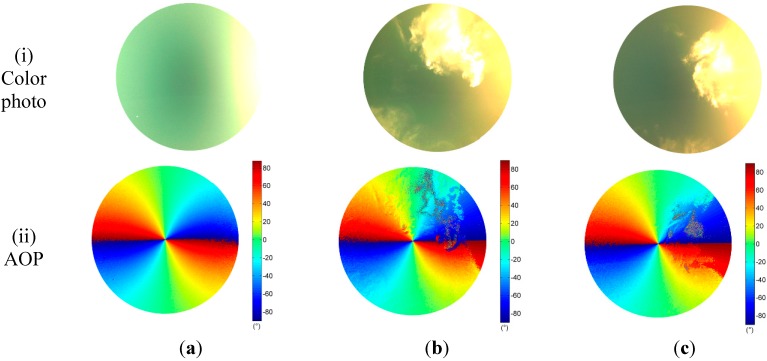

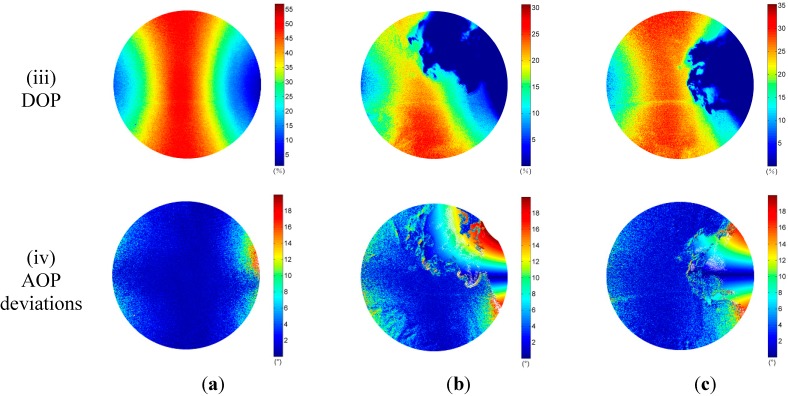
Color photo, AOP, and DOP were measured by our instrument in blue part of the spectrum in (**a**) Clear sky; (**b**) Cloudy sky I; and (**c**) Cloudy sky II. Row (iv) is the AOP deviation $\text{Δψ}=\left|{\text{ψ}}_{\text{meas}}-{\text{ψ}}_{\text{Rayleigh}}\right|$ of the three conditions.

Experiments were carried out in an outdoor environment of Changsha. Clear sky polarization patterns were performed on 8 July 2014 at 19:13, and partly cloudy sky polarization patterns were performed on 10 July 2014 at 18:50 and 19:10 in the same location. The solar elevations are 2.27°, 6.87°, and 2.81°, respectively. They were calculated by the solar position calculation algorithm in [34]. We refer to the three sky conditions simply as “clear sky”, “cloudy sky I”, and “cloudy sky II”. Patterns of DOP and AOP of both clear and cloudy skies were determined and visualized as color-coded circular maps by our system only in visible blue (470 ± 30 nm) part of the spectrum (Figure 5). Each map contains approximately 1,286,729 pixels. 

**Figure 6:**
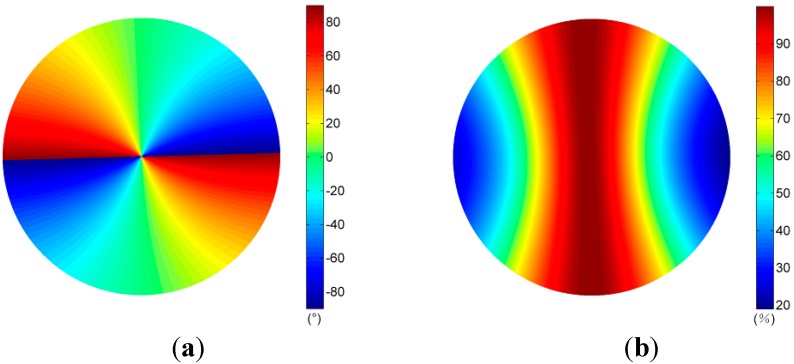
Patterns of skylight calculated by the single-scattering Rayleigh model (the position of the sun is the same as that in the clear sky). (**a**) AOP of Rayleigh sky; (**b**) DOP of Rayleigh sky.

We calculated polarization patterns of the Rayleigh sky at the same positions of the sun as those in clear and cloudy skies. Patterns of Rayleigh sky corresponding to the clear sky are shown in Figure 6. Using the measured AOP pattern of the sky in the visible blue part of the spectrum range and at three different sun positions, we calculated the AOP deviation $\text{Δψ}=\left|{\text{ψ}}_{\text{meas}}-{\text{ψ}}_{\text{Rayleigh}}\right|$ at every celestial point between the real and single-scattering Rayleigh skies, where ${\text{ψ}}_{\text{meas}}$ is the AOP of skylight measured by our full-sky imaging polarimetry, and ${\text{ψ}}_{\text{Rayleigh}}$ is the AOP of skylight calculated on the basis of the single-scattering Rayleigh model. The AOP deviations of three sky conditions are shown in row (iv) of Figure 5. 

Comparison of the measured AOP patterns (row (ii) of Figure 5) and theoretical patterns (Figure 6a) indicate that, apart from regions near the sun, anti-sun, and underneath clouds, the single-scattering Rayleigh model describe the characteristics of the sky polarization patterns relatively well. The measured AOP patterns of the clear sky follow almost the same patterns of the Rayleigh model (row (iv) of Figure 5a). 

It is noticed that the degree of polarization under cloudy skies are less than that under the clear sky (row (iii) of Figure 5). The clouds largely distort the normal distribution of the DOP as it occurs in the clear sky. They result in very low DOP patterns (row (iii) of Figure 5b,c). Furthermore, the AOP patterns in cloudy skies are also suffered distortion when clouds appear (row (ii) of Figure 5b,c, and row (iv) of Figure 5b,c. 

**Figure 7:**
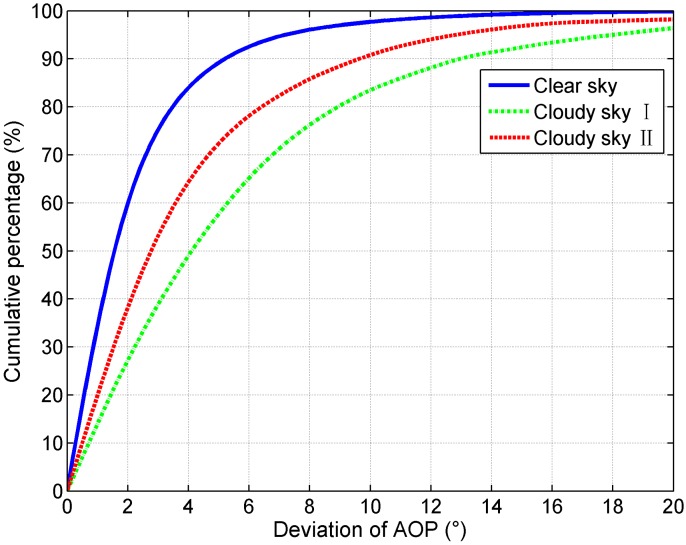
The cumulative percentages of the AOP deviations of the three sky conditions.

The cumulative percentages of the AOP deviations of three sky conditions are shown in Figure 7. In the clear sky, 84% of the deviations are less than 4°, and 60% of them are less than 2°; In the cloudy sky I, 49% of the deviations are less than 4°, and 27% of them are less than 2°; In the cloudy sky II, 64% of the deviations are less than 4°, and 38% of them are less than 2°. These results indicate that even if the clouds are present, the percentage of the sky that follows the Rayleigh model with an accuracy of Δψ≤4° is large. We can utilize the AOP patterns of skylight for orientation even on cloudy days.

However, it is undeniable that a certain percentage of data points are “non-Rayleigh” points, such as the deviations of AOP are greater than 4°. Using the “non-Rayleigh” points for orientation can significantly decrease the accuracy of the heading solution. 

It is noteworthy that the most striking AOP differences between the actual and the theoretical patterns are often present in the regions near the sun, anti-sun, neutral points and underneath clouds. These regions share a common characteristic that the degree of polarization is very low. 

**Figure 8:**
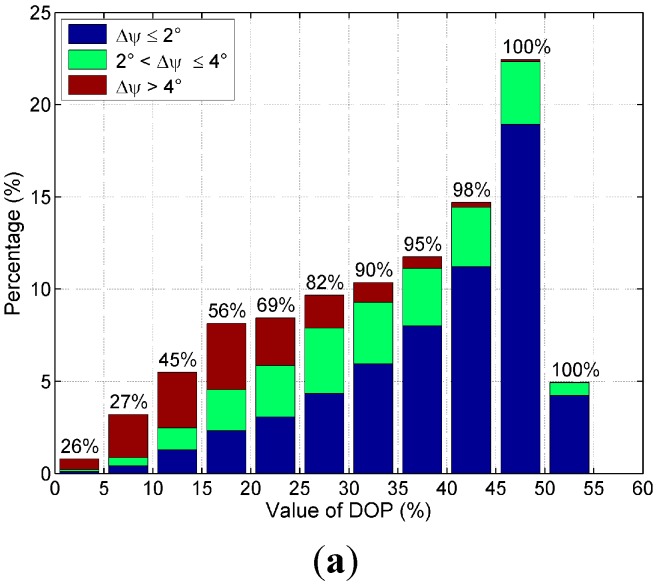

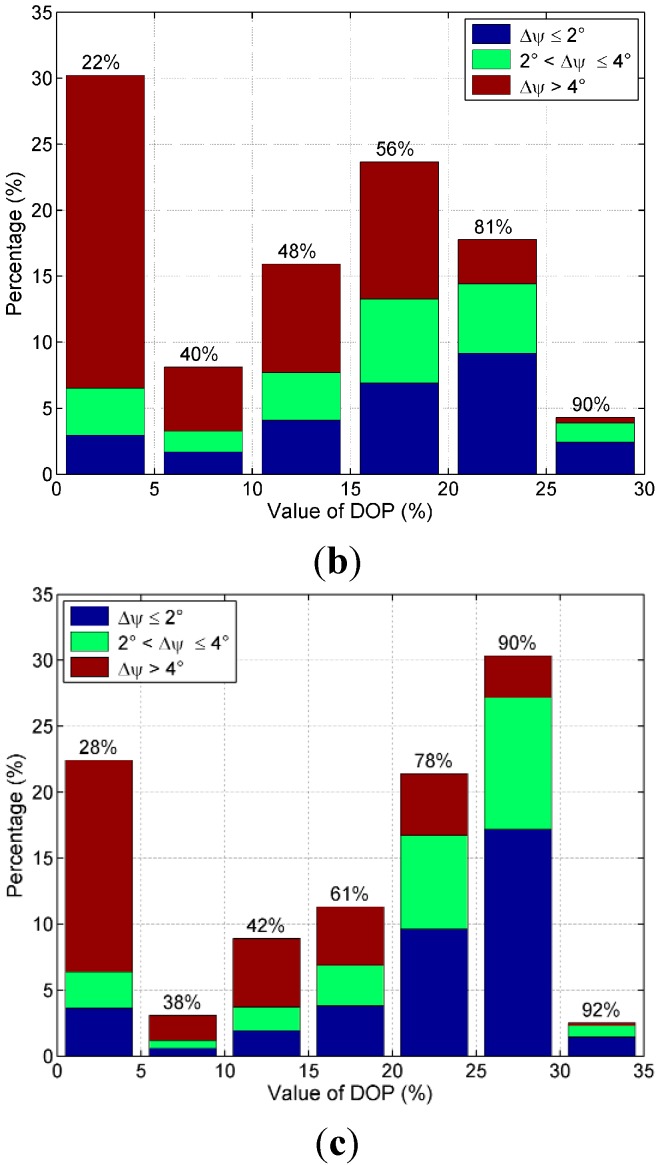
The distribution of the AOP deviations in different DOP intervals. (**a**) Clear sky; (**b**) Cloudy sky I; (**c**) Cloudy sky II.

Figure 8 shows the distribution of the AOP deviations in different intervals of DOP patterns. The proportions of the AOP deviations Δψ≤4° in each DOP interval are given on the top of the bars in the bar graphs. Analyzing the figure, we can establish the following trends: 

(1) The percentage of the sky that follows the Rayleigh model with accuracy of Δψ≤4° or Δψ≤2° in high DOP intervals is larger than that in low DOP intervals. 

(2) In various DOP intervals, the higher the DOP, the larger the percentage of the sky polarization pattern that follows the Rayleigh model in this DOP interval will be. In the clear sky, the proportion increases from 26%–100% as the DOP increases from 0 to its highest value. In the cloudy sky I, the proportion increases from 22%–90% as the DOP increases from 0 to its highest value, and from 28%–92% in the cloudy sky II as the DOP increases from 0 to its highest value. So, it will be more likely to detect the accurate AOP patterns of skylight in the region with high DOP patterns than that in the region with low DOP patterns.

**Figure 9:**
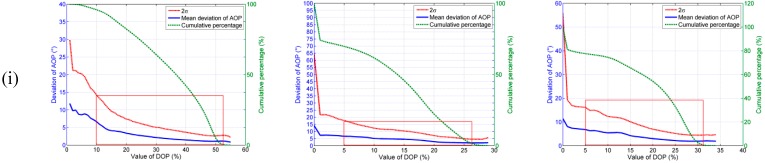

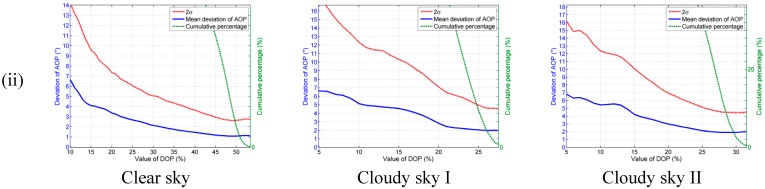
The relationships between deviations of AOP and the values of DOP (row (ii) is the drawing of partial enlargement of row (i) in the red boxes).

It can be seen from Figure 9 that the AOP deviations decrease evidently as the DOP increase from 0 to its highest value for all the three sky conditions. In the clear sky, the mean deviations of AOP can reach to 1.5° when the value of DOP is greater than 40%, and the cumulative percentage is 42%. In the cloudy sky I, the mean deviations of AOP can reach to 2.4° when the value of DOP is greater than 21%, and the cumulative percentage is 18%. In the cloudy sky II, the mean deviations of AOP are less than 2.0° when the value of DOP is greater than 26%, and the cumulative percentage is 26%. 

These results show that even if in cloudy days, we could use the AOP patterns of skylight for orientation with a high accuracy by selectively utilizing the AOP patterns in the region with high DOP patterns. 

### 4.2. Solar Meridian Orientation Calculation

We experimentally evaluate the performance of the solar meridian extraction algorithm with the AOP patterns of all the three sky conditions. Each AOP pattern contains approximately 1,286,729 pixels. The data is too large to be handled. Thus, we select the data in a circle of radius 100 pixels centered at the origin for the extraction algorithm. 

The experimental results in Section 4.1 have showed that a deviated AOP is often accompanied with a low DOP. Hence, we set the AOP patterns to zero when the corresponding DOP pattern is less than a DOP threshold *P _threshold_*. Also, the AOP measurements at the points with the same distance from the origin as those points have been set to zero. We use *P _threshold_*. = 5% for our solar meridian extraction algorithm. 

Both our method without pretreatment and the fitting based method are used for comparison. The reference heading φ is determined by an inertial navigation system (INS). It is able to yield the azimuth up to 0.01° accuracy in 10 minutes. Then, the reference solar meridian orientation θ in the b-frame can be obtained by $\text{θ}\;=\;\phi_{s}-\text{φ}$. In the experiment, the reference solar meridian orientations of the three sky conditions are −1.50°, −1.62° and −1.92°, respectively. 

**Figure 10:**
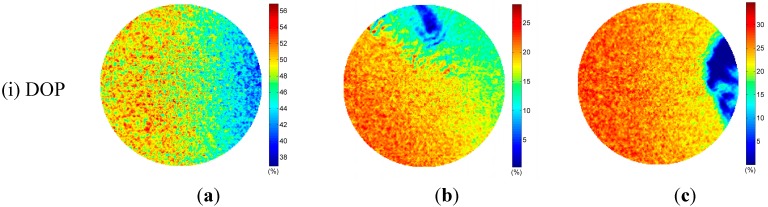

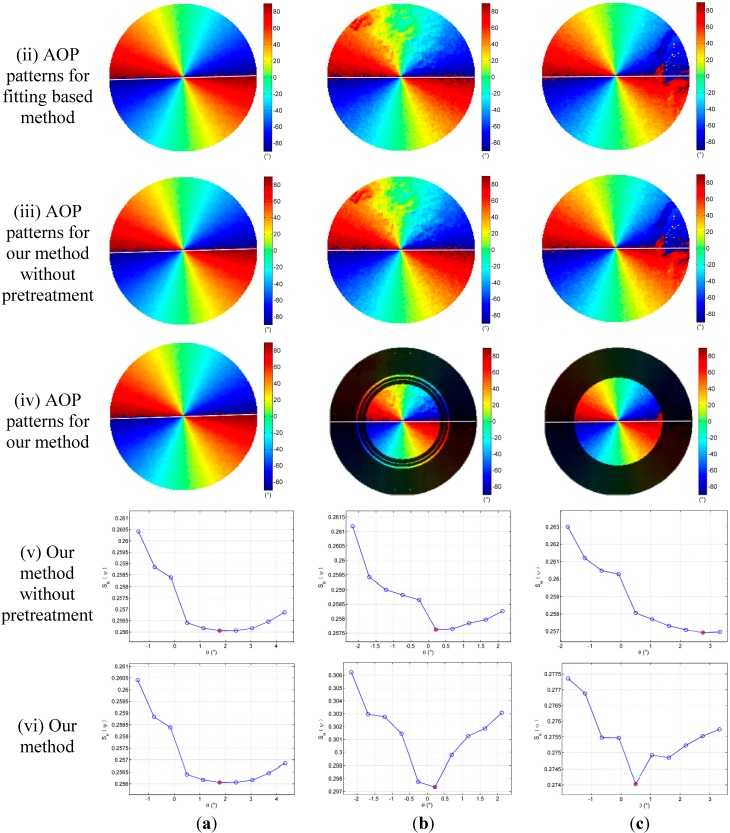
The AOP and DOP patterns in a circle of radius 100 pixels centered at the origin. (**a**) Clear sky, (**b**) Cloudy sky I, and (**c**) Cloudy sky II. (The white lines in row (ii) are the solar meridian orientations extracted by fitting based method; the white lines in row (iii) are the solar meridian orientations extracted by our method without pretreatment; the white lines in row (iv) are the solar meridian orientations extracted by our method; the black crosses in row (iv) indicate the points set to zero).

The output solar meridian orientations extracted by the three methods of the three sky conditions are shown in Figure 10. Table 3 shows the result of comparison of the solar meridian orientation errors of the three methods. We can see that our method can take full advantage of all the useful AOP measurements for the determination of the solar meridian orientations, and performs slightly better than the other two methods. All the methods can obtain stable estimates even when the clouds have great influence on the AOP patterns.

**Table 3:** The error of the solar meridian orientation extracted by the three methods

Algorithms	Clear Sky	Cloudy Sky I	Cloudy Sky II
Fitting based method	0.33°	0.48°	0.47°
Our method without pretreatment	0.27°	0.38°	1.86°
Our method	0.27°	0.38°	0.41°

It takes about 0.5 seconds in a Matlab 7.8 implementation running on a standard computer with an Intel Core 2.93 GHz processor for our solar meridian extraction method.

## 5. Discussion

Experiments in this paper were performed in three sky conditions. The results show that even if the clouds are present, the percentage of the sky that follows the Rayleigh model with a high accuracy is large. Consequently, we can utilize the AOP patterns of skylight for orientation even in cloudy days. However, the Mie and geometric pattern would be the dominant one under the condition that the atmosphere with relatively high concentrations of micro and above sized aerosols. It is noticed that the Mie scattering are dramatically different from that for the Rayleigh and geometric scattering [12,21,23]. The polarized portion of the Rayleigh scattered components are always polarized perpendicular to the scattering plane [11,12], while the polarized component of the Mie scattering results can potentially be polarized either parallel or perpendicular to the scattering plane [12,21,23]. If some of the AOP measurements are parallel to the scattering plane which is determined by the observer, the celestial point observed and the sun, the relationship between the AOP measurement and the azimuth of the sun would be uncertain. Then, it will fail to use the AOP measurement combining with the solar azimuth for orientation. Fortunately, it would be Rayleigh instead of Mie scattering even in turbid atmosphere when we use sensors to receive the polarized light that are sensitive at wavelengths longer than that which is visible [37]. It is worth researching for our future work. 

Actually, the experiments on studying the efficiency and accuracy of utilizing the AOP patterns for vehicle orientation in the paper are static tests. We need to manually rotate the linear polarizer of the polarimetry system to four different relative orientations to obtain four digital images for the calculation of polarization patterns, and cannot collect these data in real-time. Accordingly, we cannot perform the dynamic experiments by this polarimetry system. In order to resolve this problem, many real-time image based polarization sensors with different structures and/or different work principles have been developed, such as the multichannel camera-based polarization sensor [7,10,32], the CCD polarization imaging sensor [38], and the CMOS image sensor [6]. Some outdoor experiments have been done by these sensors [10,32]. Utilizing the real-time image based polarization sensor for dynamic vehicle orientation would be desirable for our future research. 

## 6. Conclusions

An evaluation of skylight AOP has been presented that compares the measurements of full-sky imaging polarimetry system with the theoretical results for vehicle orientation. Its accuracy is quantitatively evaluated in both clear and cloudy skies. The primary results demonstrate that we can utilize the angle of polarization patterns of skylight for vehicle orientation even in cloudy days, and the DOP is a key parameter to indicate the accuracy of the AOP measurement. Based on the evaluation results of the skylight polarization patterns, a solar meridian extracted method is presented. It can achieve a high accuracy for vehicle orientation on both clear and cloudy days.

## References

[1] Collett M., Collett T., Bisch S., Wehner R. (1998). Local and global vectors in desert ant navigation. Nature.

[2] Shashar N., Johnsen S., Lerner A., Sabbah S., Chiao C.-C., Mathger L.M., Hanlon R.T. (2011). Underwater linear polarization: Physical limitations to biological functions. Phil. Trans. R. Soc. B.

[3] Horváth G., Varju D. (2004). Polarized light in animal vision: Polarization patterns in nature.

[4] Lambrinos D., Möller R., Labhart T., Pfeifer R., Wehner R. (2000). A mobile robot employing insect strategies for navigation. Robot. Auton. Syst..

[5] Chu J., Zhao K., Zhang Q., Wang Tichang (2008). Construction and performance test of a novel polarization sensor for navigation. Sensor. Actuat. A-Phys..

[6] Sarkar M., Theuwissen A. (2013). A Biologically Inspired cmos Image Sensor.

[7] Horvath G., Barta A., Gal J., Suhai B., Haiman O. (2002). Ground-based full-sky imaging polarimetry of rapidly changing skies and its use for polarimetric cloud detection. Appl. Opt..

[8] Chahl J., Mizutani A. (2012). Biomimetic attitude and orientation sensors. IEEE Sensor. J..

[9] Karman S.B., Diah S.Z.M., Gebeshuber I.C. (2012). Bio-inspired polarized skylight-based navigation sensors: A review. Sensors.

[10] Sturzl W., Carey N. A Fisheye Camera System for Polarisation Detection on Uavs. Proceedings of Computer Vision-ECCV 2012 Workshops and Demonstrations.

[11] Pomozi I., Horvath G., Wehner R. (2001). How the clear-sky angle of polarization pattern continues underneath clouds: Full-sky measurements and implications for animal orientation. J. Exp. Biol..

[12] Pust N.J., Shaw J.A. (2008). Digital all-sky polarization imaging of partly cloudy skies. Appl. Opt..

[13] Miyazaki D., Ammar M., Kawakami R., Ikeuchi K. (2008). Estimating sunlight polarization using a fish-eye lens. IPSJ J..

[14] Suhai B., Horvath G. (2004). How well does the rayleigh model describe the e-vector distribution of skylight in clear and cloudy conditions? A full-sky polarimetric study. J. Opt. Soc. Am. A.

[15] Kreuter A., Zangerl M., Schwarzmann M., Blumthaler M. (2009). All-sky imaging: A simple, versatile system for atmospheric research. Appl. Opt..

[16] Raymond L., Lee J., Samudio O.R. (2012). Spectral polarization of clear and hazy coastal skies. Appl. Opt..

[17] Kreuter A., Blumthaler M. (2013). Feasibility of polarized all-sky imaging for aerosol characterization. Atmos. Meas. Tech..

[18] Horvath G., Varju D. (1995). Underwater refraction-polarization patterns of skylight perceived by aquatic animals through snell's wiindow of the flat water surface. Vision Res..

[19] Sabbah S., Lerner A., Erlick C., Shashar N. (2005). Under water polarization vision-a physical examination. Recent Res. Devel. Exp. Theor. Biol..

[20] Sabbah S., Barta A., Gál J., Horváth G., Shashar N. (2006). Experimental and theoretical study of skylight polarization transmitted through snell’s window of a flat water surface. J. Opt. Soc. Am. A.

[21] Lerner A., Sabbah S., Erlick C., Shashar N. (2011). Navigation by light polarization in clear and turbid waters. Philos. Tran. R. Soc. B: Biol. Sci..

[22] You Y., Tonizzo A., Gilerson A.A., Cummings M.E., Brady P., Sullivan J.M., Twardowski M.S., Dierssen H.M., Ahmed S.A., Kattawar G.W. (2011). Measurements and simulations of polarization states of underwater light in clear oceanic waters. Appl. Opt..

[23] Lerner A., Shashar N., Haspel C. (2012). Sensitivity study on the effects of hydrosol size and composition on linear polarization in absorbing and nonabsorbing clear and semi-turbid waters. J. Opt. Soc. Am. A.

[24] Brines M.L., Gould J.L. (1982). Skylight polarization patterns and animal orientation. J. Exp. Biol..

[25] Gal J., Horvath G., Meyer-Rochow V.B., Wehner R. (2001). Polarization patterns of the summer sky and its neutral points measured by full-sky imaging polarimetry in finnish lapland north of the arctic circle. Proc. R. Soc. Lond. A.

[26] Henze M.J., Labhart T. (2007). Haze, clouds and limited sky visibility: Polarotactic orientation of crickets under difficult stimulus conditions. J. Exp. Biol..

[27] Raymond L., Lee J. (1998). Digital imaging of clear-sky polarization. Appl. Opt..

[28] Calibration Toolbox for Matlab. http://www.vision.caltech.edu/bouguetj/calib_doc/index.html.

[29] Wang Y., Hu X., Lian J., Zhang L., Xian Z., Ma T. (2014). Design of a device for skylight polarization measurements. Sensors.

[30] Voss K.J., Liu Y. (1997). Polarized radiance distribution measurements of skylight. I. System description and characterization. Appl. Opt..

[31] Goldstein D. (2003). Polarized Light.

[32] Wang D., Liang H., Zhu H., Zhang S. (2014). A bionic camera-based polarization navigation sensor. Sensors.

[33] Berry M.V., Dennis M.R., Lee R.L. (2004). Polarization singularities in the clear sky. New J. Phys..

[34] Blanco-Muriel M., Alarcon-Padilla D.C., Lopezmoratalla T., Lara-coira M. (2001). Computing the solar vector. Sol. Energy.

[35] Kiryati N., Gofman Y. (1998). Detecting symmetry in grey level images: The global optimization approach. Int. J. Comput. Vision.

[36] Shabayek A.E.R. (2012). Combining Omnidirectional Vision with Polarization Vision for Robot Navigation. Ph.D. Thesis.

[37] Bohren C.F., Huffman D.R. (1983). Absorption and Scattering of Light by Smalll Particles.

[38] Gruev V., Perkins R., York T. (2010). CCD polarization imaging sensor with aluminum nanowire optical filters. Opt. Express.

